# Serum Metabolomic Profiling Reveals Biomarkers for Early Detection and Prognosis of Esophageal Squamous Cell Carcinoma

**DOI:** 10.3389/fonc.2022.790933

**Published:** 2022-01-28

**Authors:** Pan Pan Wang, Xin Song, Xue Ke Zhao, Meng Xia Wei, She Gan Gao, Fu You Zhou, Xue Na Han, Rui Hua Xu, Ran Wang, Zong Min Fan, Jing Li Ren, Xue Min Li, Xian Zeng Wang, Miao Miao Yang, Jing Feng Hu, Kan Zhong, Ling Ling Lei, Liu Yu Li, Yao Chen, Ya Jie Chen, Jia Jia Ji, Yuan Ze Yang, Jia Li, Li Dong Wang

**Affiliations:** ^1^ State Key Laboratory of Esophageal Cancer Prevention & Treatment and Henan Key Laboratory for Esophageal Cancer Research of the First Affiliated Hospital, Zhengzhou University, Zhengzhou, China; ^2^ Department of Oncology, The First Affiliated Hospital of Henan University of Science and Technology, Luoyang, China; ^3^ Department of Thoracic Surgery, Anyang Tumor Hospital, Anyang, China; ^4^ Department of Pathology, The Second Affiliated Hospital of Zhengzhou University, Zhengzhou, China; ^5^ Department of Pathology, Hebei Provincial Cixian People’s Hospital, Cixian, China; ^6^ Department of Thoracic Surgery, Linzhou People’s Hospital, Linzhou, China

**Keywords:** biomarkers, metabolic profiles, early detection, esophageal carcinoma, prognosis

## Abstract

Esophageal squamous cell carcinoma (ESCC) is one of the most common aggressive malignancies worldwide, particularly in northern China. The absence of specific early symptoms and biomarkers leads to late-stage diagnosis, while early diagnosis and risk stratification are crucial for improving overall prognosis. We performed UPLC-MS/MS on 450 ESCC patients and 588 controls consisting of a discovery group and two validation groups to identify biomarkers for early detection and prognosis. Bioinformatics and clinical statistical methods were used for profiling metabolites and evaluating potential biomarkers. A total of 105 differential metabolites were identified as reliable biomarker candidates for ESCC with the same tendency in three cohorts, mainly including amino acids and fatty acyls. A predictive model of 15 metabolites [all-trans-13,14-dihydroretinol, (±)-myristylcarnitine, (2S,3S)-3-methylphenylalanine, 3-(pyrazol-1-yl)-L-alanine, carnitine C10:1, carnitine C10:1 isomer1, carnitine C14-OH, carnitine C16:2-OH, carnitine C9:1, formononetin, hyodeoxycholic acid, indole-3-carboxylic acid, PysoPE 20:3, PysoPE 20:3(2n isomer1), and resolvin E1] was developed by logistic regression after LASSO and random forest analysis. This model held high predictive accuracies on distinguishing ESCC from controls in the discovery and validation groups (accuracies > 89%). In addition, the levels of four downregulated metabolites [hyodeoxycholic acid, (2S,3S)-3-methylphenylalanine, carnitine C9:1, and indole-3-carboxylic acid] were significantly higher in early cancer than advanced cancer. Furthermore, three independent prognostic markers were identified by multivariate Cox regression analyses with and without clinical indicators: a high level of MG(20:4)isomer and low levels of 9,12-octadecadienoic acid and L-isoleucine correlated with an unfavorable prognosis; the risk score based on these three metabolites was able to stratify patients into low or high risk. Moreover, pathway analysis indicated that retinol metabolism and linoleic acid metabolism were prominent perturbed pathways in ESCC. In conclusion, metabolic profiling revealed that perturbed amino acids and lipid metabolism were crucial metabolic signatures of ESCC. Both panels of diagnostic and prognostic markers showed excellent predictive performances. Targeting retinol and linoleic acid metabolism pathways may be new promising mechanism-based therapeutic approaches. Thus, this study would provide novel insights for the early detection and risk stratification for the clinical management of ESCC and potentially improve the outcomes of ESCC.

## Introduction

Esophageal cancer is the eighth most common form of cancer and the sixth leading cause of cancer death in the world (1). Esophageal squamous cell carcinoma (ESCC) remains the predominant histological type globally (accounts for 90%), especially in northern China (2). Due to occult symptoms in early stage, 80% of ESCC patients are in the middle or advanced stage at the time of diagnosis, with a 5-year survival of only 20% (3). Therefore, identifying phenotypic characteristics and predictive biomarkers are of great significance for the early detection and improvement of the prognosis of ESCC.

Metabolomics has emerged as a new high-throughput “omics” technology for screening low molecular weight metabolites (<1,000 Da) in biological samples, which can directly reflect the pathological state after gene mutation and/or protein variations (4, 5). It has been generally accepted that cancer is a metabolic disease with metabolic reprogramming (6). Hitherto, metabolomics has been used to examine global metabolite profiles and screen biomarkers for early warning and monitoring of multiple cancers (7–10), as well as to further gain insight into the potential mechanisms of tumorigenesis and progression of cancers (11).

In recent years, the exploration of metabolic characteristics and diagnostic and prognostic markers for esophageal cancer has attracted much attention. For instance, Wang et al. showed that 16 biomarkers as ESCC-related metabolic signatures could be used for diagnosis, among which dodecanoic acid, LysoPA (18:1), and LysoPC (14:0) could be markers of disease progression (12). Another study constructed an effective diagnostic model based on eight metabolites consisting of hypoxanthine, proline betaine, indoleacrylic acid, inosine, 9-decenoylcarnitine, tetracosahexaenoic acid, LPE (20:4), and LPC (20:5) and found that indoleacrylic acid, LPC (20:5), and LPE (20:4) had association with ESCC progression (13). One research by Chen et al. showed that four circulating metabolites, kynurenine, 1-myristoyl-glycero-3-phosphocholine [LPC (14:0) sn-1], 2-piperidinone, and hippuric acid, acted as potential ESCC prognostic biomarkers (14). However, due to dynamic and sensitive features of the metabolome, these studies may be limited by small sample size or lack of validation groups; thus, our understanding of metabolism-related changes in esophageal cancer remains limited.

We therefore did this multicenter, large-scale cohort study to determine global alterations of metabolites and screen biomarkers. We performed a widely targeted metabolome by UPLC-MS/MS on serum samples of 450 ESCC patients and 588 controls consisting of the discovery group (training set and test set) and two validation groups to identify biomarkers for early detection. The relationships between these biomarkers and tumor stage were further explored. In addition, clinicopathological indicators and metabolites were integrated to identify molecular markers with prognostic value. Differentially expressed metabolites in tissues provided further validation of these markers from serum. Biomarkers for early detection will facilitate and supplement the criteria of high-risk population of esophageal cancer, improving the efficiency of non-invasive detection and monitoring. Meanwhile, prognostic biomarkers potentially shed new light on risk stratification and management for patients with esophageal cancer. Finally, pathway analysis based on these findings could contribute to uncovering pathogenetic mechanism and identifying potential therapeutic targets.

## Materials and Methods

### Participants and Sample Collection

A total of 1,038 cases (450 ESCC patients and 588 healthy controls) were recruited from multicenter esophageal and gastric cardia carcinoma databases (1975–2021) established by the State Key Laboratory for Esophageal Cancer Prevention & Treatment and Henan Key Laboratory for Esophageal Cancer Research of the First Affiliated Hospital of Zhengzhou University. Detailed clinicopathological data and qualified blood samples were available for all participants.

Three hundred and seventy-one patients were enrolled from September 2013 to January 2020, divided into two groups: 225 ESCC patients (November 2019 to February 2020) as the discovery set and 146 cases (September 2013 to October 2019) as the verification set 1. All clinicopathological features of patients were extracted from medical records, including age, gender, family history, tumor site, T stage, N stage, and TNM stage. An independent group of 79 patients as another external verification set was obtained from high incidence areas of esophageal cancer in China during clinical epidemiological investigation. Clinical and pathological data were collected by questionnaires and hospital information system retrospectively. For healthy controls, 588 individuals were enrolled after excluding any upper gastrointestinal tumors *via* gastroscopic biopsy between 2012 and 2020, and they were randomly selected and matched into the discovery group and verification groups with 363, 165, and 60, respectively.

Fasting blood samples of patients in the discovery set and verification set 1 were collected before surgery during hospitalizations, and those of verification set 2 and healthy controls were taken during site investigation and gastrointestinal endoscopy, respectively. All samples of cases and controls were drawn into blood non-anticoagulant tube, standing at room temperature for 30 min awaiting natural coagulation. After centrifugation at 12,000 r/min for 3 min, the supernatant fraction was collected and divided into equal parts (0.5 ml) and then stored in a refrigerator at −80°C until further analysis.

Three pairs of tissues (tumor and adjacent normal samples) obtained from the same patients in the discovery group were used for further validation of serum biomarkers. Tissue samples were collected within 30 min after operation, frozen in liquid nitrogen, and stored at −80°C. The study design and procedures are presented in **Figure S1**.

All patients were confirmed as esophageal cancer by two pathologists independently. Familial history was considered positive if the proband had one or more cancer-affected relatives in three consecutive generations. Regions with ESCC incidence over 60/10 million were classified as high incidence areas of esophageal cancer, and low incidence areas otherwise. TNM staging was performed according to the sixth Union for International Cancer Control (UICC) TNM classification system due to the large span of diagnosis. Stages I and IIA were defined as early cancer, and stages IIB, III, and IV as advanced cancer. The follow-up for overall survival was *via* telephone or home investigation every 3–6 months. Each participant signed the informed consent form, and ethical approval for this study was obtained from the Medical Ethics Committee of the First Affiliated Hospital of Zhengzhou University.

### Serum Pretreatment

Serum samples were removed from −80°C and thawed on ice immediately until thawing completely. After vortexed for 10 s, 50 μl of each sample was transferred to a centrifuge tube with the corresponding number, mixed with 300 μl pure methanol, then whirled for 3 min, and centrifugated at 12,000 r/min at 4°C for 10 min. The supernatant (200 μl) was absorbed to a new centrifuge tube, followed by standing at −20°C for 30 min. After centrifuged at 12,000 r/min for 3 min at 4°C, 150 μl of the supernatant was taken to the corresponding injection bottle for metabolomic analysis.

### Tissue Pretreatment

Tissue was taken out from −80°C and kept on ice throughout the process. Thawed tissue was minced and 20 mg of sample was weighed by multi-point sampling then transferred into a centrifuge tube to homogenize (30 Hz) for 20 s with a steel ball. After centrifugation at 3,000 r/min, 4°C for 30 s, the pellet was added into 400 μl of 70% methanol water internal standard extractant with shaking (1,500 r/min) for 5 min and then kept on ice for 15 min. The supernatant (200 μl) was recovered after centrifugation (12,000 r/min, 10 min, 4°C) and then was allowed to stand at −20°C for 30 min. After centrifugation (12,000 r/min, 4°C) for 3 min, 200 μl of supernatant was collected for analysis.

### UPLC-MS/MS Analysis

Metabolomics analysis on serum and tissue was performed using an LC-ESI-MS/MS system (Ultra Performance Liquid Chromatography, UPLC, ExionLC AD, https://sciex.com.cn/; tandem mass spectrometry, MS/MS, QTRAP^®^ System, https://sciex.com/). The UPLC conditions included chromatographic column (Waters ACQUITY UPLC HSS T3 C18, 1.8 µm, 2.1 mm * 100 mm) and mobile phase A (ultrapure water containing 0.1% formic acid) and B (acetonitrile containing 0.1% formic acid). The elution gradient was as follows: mobile phase A/B (95:5 V/V) at 0 min, 10:90 V/V at 10.0 min, 10:90 V/V at 11.0 min, 95:5 V/V at 11.1 min, and 95:5 V/V at 14.0 min. The injection was 2 μl at a flow rate of 0.4 ml/min with column temperature of 40°C.

The MS/MS depended on a triple quadrupole linear ion trap mass spectrometer (QTRAP), QTRAP^®^ LC-MS/MS System, used to do the linear ion trap (LIT) and triple quadrupole (QQQ) scans. The parameters of QTRAP^®^ LC-MS/MS System were set as follows: electrospray ionization (ESI) source temperature, 500°C; ion spray voltage (IS), −4,500 to 5,500 V; ion source gas I (GSI), 55 psi; GSII, 60 psi; curtain gas (CUR), 25 psi; and collision gas (CAD), high level. Polypropylene glycol solutions (100 and 10 μmol/L) were used for instrument tuning and mass calibration in LIT and QQQ modes, respectively. Here, each ion pair was scanned according to the declustering potential (DP) and collision energy (CE) through multiple reaction monitoring (MRM).

Sample extract mixtures as quality controls (QCs) were inserted into every 10 testing samples to monitor the repeatability of the analysis process.

### Data Processing, Quality Control, and Statistical Analyses

After MS data were analyzed with software Analyst 1.6.3 (AB SCIEX, Ontario, Canada), qualitative analysis of metabolites was done according to retention time (RT), ion pair, and second spectra, based on MetWare database (http://www.metware.cn/) and publicly available metabolite databases, such as HMDB (http://hmdb.ca/) and MassBank (http://www.massbank.jp/). The quantitative analysis steps were as follows: first, screening the characteristic ions of each substance by triple quadrupole, we obtained the signal strength of characteristic ions in the detector. Then through integrating and correcting chromatographic peaks by the software MultiQuant, we obtained the relative content of the corresponding substance represented by the area of each chromatographic peak. Metabolite annotation was performed based on the KEGG compound database (http://www.kegg.jp/kegg/compound/).

Data quality was assessed using principal component analysis (PCA, princomp function in R) and coefficient of variation value (CV: ratio of standard deviation to mean, Microsoft Excel 2016 and ggplot2 in R). These results supported the reliability of data—that QC samples clustered together clearly in the PCA plots of the three groups and more than 85% metabolites with CV values were less than 15% (**Figure S2**).

Multivariate statistical investigations were performed including PCA and orthogonal partial least squares-discriminant analysis (OPLS-DA, ropls package in R). Model quality of OPLS-DA was estimated by R2Y and Q2 values. Volcano plots were generated based on log2 fold changes and −log10 (*p*-values) by R. Heatmaps were performed and visualized with pheatmap package. Statistically significant metabolites were selected by *p <*0.05 (Student’s *t*-test or Wilcoxon test), and variable importance in the projection (VIP) generated from the OPLS-DA model was referenced as supplement.

Least absolute shrinkage and selection operator (LASSO) regression model and random forest were performed to screen potential biomarkers for diagnosing ESCC (glmnet package and randomforest package). The logistic regression model was trained using the function glm in R. Receiver operating characteristic curves (ROC) were plotted to evaluate the predictive accuracy of the diagnostic model based on metabolites with GraphPad software. Violin plots produced with ggviolin package were used to visualize differences in metabolites between early and advanced cancer patients based on the Wilcoxon test.

Kaplan–Meier survival curves and log-rank test were used to calculate survival rate and compared survival curves between groups, respectively (survival and survminer package). Cox proportional hazards regression test was carried out to analyze prognostic factors for overall survival and compute hazard ratio (HR) and 95% confidence interval (CI) of multivariate survival analysis (survival package). Moreover, forest plots were generated with the forestplot package based on the results of Cox regression analyses. Correlations between serum and tissue metabolites were assessed by Pearson’s correlation coefficients using R function cor, and the network diagram was visualized in Cytoscape (version 3.8.0). Pathway analysis was undertaken with MetaboAnalyst 5.0 online software using the KEGG pathway database.

Statistical analyses were conducted using R software (version 4.0.4) and Prism8 (GraphPad) software, and *p <*0.05 was considered statistically significant.

## Results

### Clinical Characteristics and Metabolomic Study of ESCC

To explore the metabolomic profiles and biomarker candidates for ESCC, 1,038 participants (450 patients and 588 normal individuals) were enrolled: the discovery cohort consisted of 588 (225 patients and 363 controls), while the two validation groups consisted of 311 and 139 (146 patients and 165 controls and 79 patients and 60 controls, respectively). Their clinical characteristics are shown in **Table S1**.

**Figure 1:**
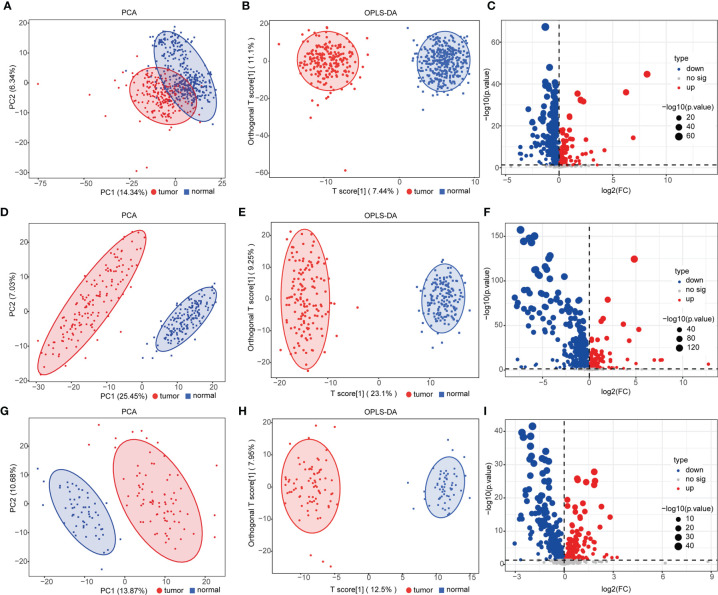
Analysis of serum metabolomics of patients with esophageal squamous cell carcinoma (ESCC) and controls. **(A, D, G)** Principal component analysis (PCA) of metabolomic data from ESCC (red) and normal (blue) samples in the discovery group **(A)** and validation groups 1 **(D)** and 2 **(G)**. **(B, E, H)** Orthogonal partial least squares-discriminant analysis (OPLS-DA) score plot of all detected metabolites [**(B)** discovery group, R2X = 0.295, R2Y = 0.949, Q2 = 0.918; **(E)** validation group 1, R2X = 0.323, R2Y = 0.976, Q2 = 0.973; **(H)** validation group 2, R2X = 0.279, R2Y = 0.968, Q2 = 0.942] in ESCC (red) and normal (blue) groups. **(C, F, I)** Volcano plot [−log10(*p*-value) and log2(fold-change)] depicting upregulated (red) and downregulated (blue) metabolites in ESCC with *p <*0.05 in the discovery group **(C)** and validation groups 1 **(F)** and 2 **(I)**. See also **Tables S3–S5**.

We performed UPLC-MS/MS analysis in serum samples of all subjects to profile the entire metabolome of esophageal cancer. Qualitative and quantitative analyses of metabolite levels were performed based on metabolic databases. A total of 963 metabolites were finally annotated (**Table S2**). In the discovery set, 524 compounds (155 upregulated and 369 downregulated metabolites) were with significant difference between ESCC and controls (*p* < 0.05) (**Table S3**). PCA and OPLS-DA were applied to characterize the metabolic patterns of ESCC, exhibiting a clear separation between ESCC and normal (**Figures 1A, B** **)**. The OPLS-DA model had R2Y at 0.949 and Q2 at 0.918. These differential metabolites were further validated in two independent groups. Similarly, PCA and OPLS-DA of the two validation sets also showed clear clusters of patients and controls (**Figures 1D, E, G, H** **)**. The models developed by OPLS-DA had high fitness and prediction values (R2Y = 0.976 and Q2 = 0.973, R2Y = 0.968 and Q2 = 0.942, respectively). Among these metabolites, 382 metabolites were validated with statistically significant difference (*p* < 0.05) between ESCC and controls in validation set 1 and 248 in validation set 2 (**Tables S4, S5**). Volcano plots showed the distributions of both downregulated and upregulated metabolites for ESCC (**Figures 1C, F, I** **)**.

**Figure 2:**
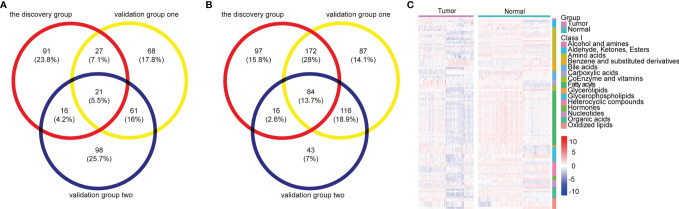
Metabolomic profiles of patients with ESCC compared with controls. **(A, B)** Venn diagram of differential metabolites overlaps from the discovery and validation groups: 21 upregulated **(A)** and 84 downregulated **(B)** differential metabolites validated as reliable biomarker candidates with the same trend. **(C)** Heatmap summarizing differential metabolites in ESCC patients versus normal controls based on metabolite classes. Color bars at the right from top to down indicate samples from patients (purple) and controls (green), metabolites classes, and relative abundance of upregulated (red) and downregulated (blue) metabolites, respectively. Each column represents an individual subject, and each row a metabolite. See also **Table S6**.

Ultimately, a total of 105 differential metabolites (21 upregulated and 84 downregulated metabolites) were validated as reliable biomarker candidates maintaining the same tendency as the discovery set (**Figures 2A, B** and **Table S6**). Twenty-one upregulated metabolites mainly included four glycerophospholipids, three sugar alcohols, three benzene and substituted derivatives, two aldehydes, two oxidized lipids, and two nucleotides and its metabolites. Meanwhile, the major categories of 84 downregulated metabolites were fatty acyls, amino acids, indoles, bile acids, organic acids, oxidized lipids, and glycerophospholipids. We noted that almost all serum amino acid-related (alanine, histidine, glycine, serine, phenylalanine, cysteine, isoleucine) and lipid-related metabolites (acylcarnitine, fatty acids) were decreased, lysophosphatidylcholine (LPC) was upregulated, and lysophosphatidylethanolamine (LPE) was downregulated, while benzene and substituted derivatives as external environmental factors were upregulated. Metabolomic data were also visualized using heatmaps with metabolites arranged by major classes (**Figure 2C**). In summary, the global alterations of the metabolic profile for ESCC were significantly different compared with normal groups.

### Metabolite Diagnostic Biomarkers for ESCC

**Figure 3:**
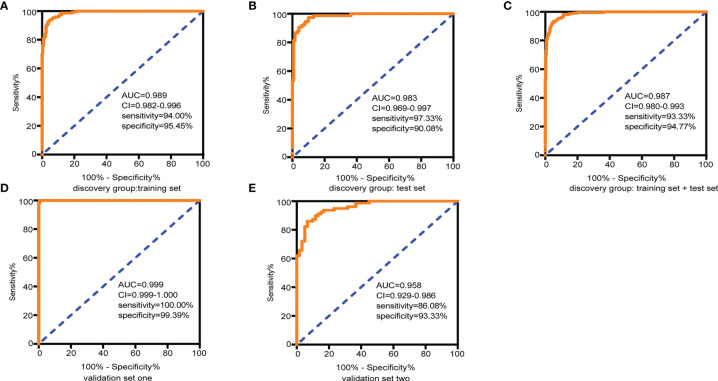
Diagnostic capacity of the combined 15 selected metabolite biomarkers in discrimination of ESCC and controls assessed by receiving operator characteristic (ROC) curves. **(A)** Training set, 150 tumors and 242 normal samples; **(B)** test set, 75 tumors and 121 normal samples; **(C)** the discovery group (training set + test set), 225 tumors and 363 normal samples; **(D)** validation group 1, 146 tumors and 165 normal samples; and **(E)** validation group 2, 79 tumors and 60 normal samples. Abbreviations: AUC, area under the curve in the ROC curves; 95% CI, 95% confidence interval. See also **Table S7**.

**Figure 4:**
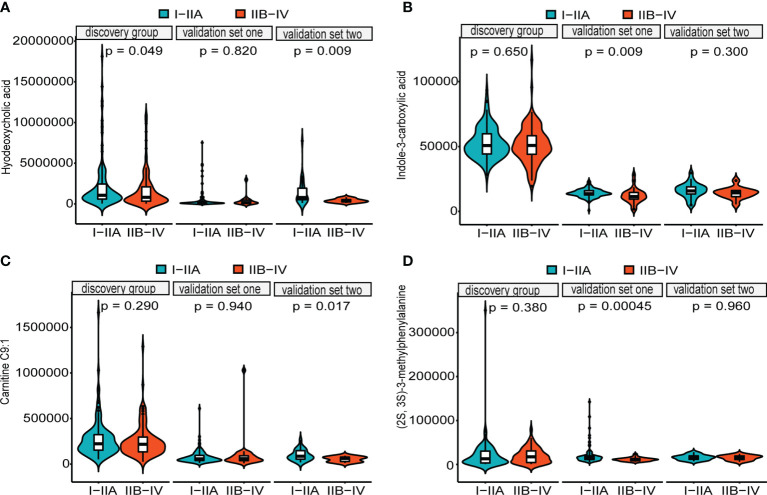
Distributions of five metabolites between early cancer and advanced cancer. Violin plots depicting the levels of hyodeoxycholic acid **(A)**, indole-3-carboxylic acid **(B)**, carnitine C9:1 **(C)**, and (2S,3S)-3-methylphenylalanine **(D)**. All of them were significantly higher in early cancer (I–IIA stage) (green) than in advanced cancer (IIB–IV stage) (orange) patients with ESCC (*p* < 0.05, Wilcoxon test). Internal box plots represent median and interquartile range of metabolites across patients.

LASSO regression and random forest were performed in the discovery groups to further identify metabolite biomarkers for distinguishing ESCC patients from the healthy population. All quantitative data of metabolites were analyzed after normalization and logarithm transformed. Individuals including ESCC patients and healthy controls in the discovery group were divided randomly into training set (150 patients and 242 controls) and test set (75 patients and 121 controls) at a ratio of 2:1. A 10-fold cross-validation was used to estimate the optimal parameter (lambda) of the model and select the optimal combination of variables. Fifteen metabolites were selected as the most important predictors by combinations of two methods, including all-trans-13,14-dihydroretinol (upregulation) and (±)-myristylcarnitine, (2S,3S)-3-methylphenylalanine, 3-(pyrazol-1-yl)-L-alanine, carnitine C10:1, carnitine C10:1 isomer1, carnitine C14-OH, carnitine C16:2-OH, carnitine C9:1, formononetin, hyodeoxycholic acid, indole-3-carboxylic acid, PysoPE 20:3, PysoPE 20:3(2n isomer 1), and resolvin E1 (downregulation) (**Table S7**). We constructed and trained this metabolite-based model using logistic regression analysis showing high diagnostic performance for ESCC: an accuracy of 94.9% in the training set, 92.57% in the test set, and 94.22% in the entire discovery group, with area under the ROC curves (AUC) greater than 0.98 (**Figures 3A–C** and **Table 1**). To test the generalizability of this panel, we did same analyses in the two independent validation cohorts. Fortunately, we got similar results as the discovery set (**Figures 3D, E** **)**. Additionally, the levels of four downregulated metabolites [hyodeoxycholic acid, (2S,3S)-3-methylphenylalanine, carnitine C9:1, and indole-3-carboxylic acid] were significantly higher in I–IIA stage (early cancer) than in IIB–IV stage (advanced cancer) (*p* < 0.05, Wilcoxon test) (**Figures 4A–D**). In conclusion, this combination of 15 serum metabolites could be used in different populations as reliable potential biomarkers for the early detection of esophageal cancer.

**Table 1:** Performance for ESCC diagnosis of metabolite biomarkers on the training set and validation sets.

Study and model	No. of subjects			Sensitivity (%)	Specificity (%)	Accuracy (%)	AUC	95% CI
	ECSS	Healthy controls	Total					
Discovery group: training set	150	242	392	94.00	95.45	94.90	0.989	0.982–0.966
Discovery group: test set	75	121	196	97.33	90.08	92.57	0.983	0.969–0.997
Discovery group: training set + test set	225	363	588	93.33	94.77	94.22	0.987	0.980–0.993
Validation set 1	146	165	311	100.00	99.39	99.68	0.999	0.999–1.000
Validation set 2	79	60	139	86.08	93.33	89.21	0.958	0.929–0.986

AUC, area under the ROC curve; 95% CI, 95% confidence interval.

### Prognostic Metabolic Biomarkers for ESCC

**Figure 5:**
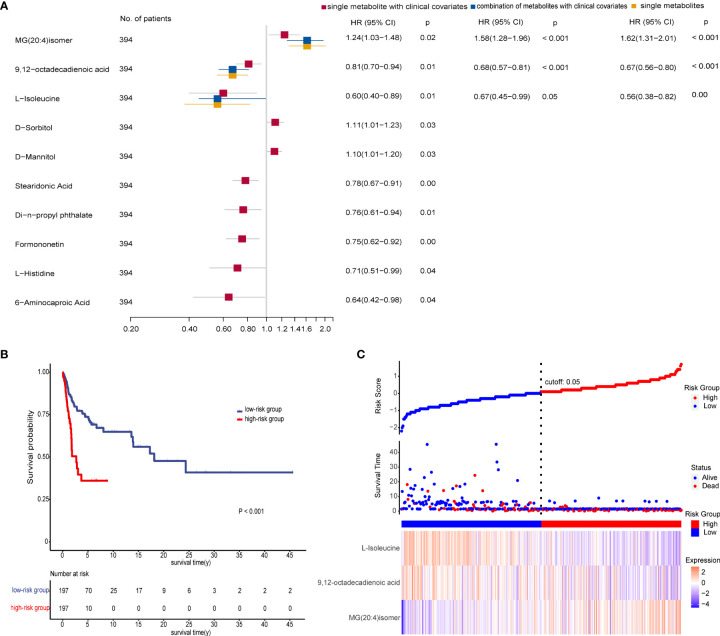
Prognostic value of the metabolite-based model from various multivariate Cox regression analyses on all patients. **(A)** Forest plot for multivariate Cox regression model of biomarkers with and without clinical factors associated with overall survival (OS) of patients (*n* = 394). Hazard ratio (HR) is depicted on the *x*-axis and each prognostic variable on the *y*-axis. HR >1 indicates poor prognosis. Black dots indicate point estimates, the horizontal lines represent the 95% CIs, and the vertical dashed line represents the reference line for an HR of 1. **(B)** Kaplan–Meier plots displaying the significant difference of overall survival in two patient groups (*p* < 0.001). The risk score of the Cox regression model stratified patients into low-risk (blue) and high-risk (red) groups according to median. Numbers of survivors at different time points stratified by risk group are shown in the table below. **(C)** Risk score analysis consisting of three parts: up part, risk score distribution of the low-risk (blue) and high-risk (red) groups (up); middle part, scatter plots representing the survival status and duration of ESCC cases; down part, heatmap of the relative expression levels of MG(20:4)isomer, 9,12-octadecadienoic acid, and L-isoleucine. See also **Table S8**.

We performed Kaplan–Meier log-rank tests and univariate and multivariate Cox regression analyses on all patients in the three groups to identify prognostic factors. Results revealed that 19 metabolites had statistical significance in both survival analysis by a median-split (*p* < 0.05) and univariate Cox regression analyses (*p* < 0.2) (**Table S8**). After excluding four metabolites that affected the survival rate with time through proportional hazards assumption (PH), each metabolite was further subjected to multivariate Cox regression analysis adjusted for clinical covariates (age, TNM stage, N stage, family history, and high or low incidence areas) separately. These observations demonstrated that 10 metabolites were significantly associated with overall survival. Then multivariate stepwise Cox regression analyses with backward elimination were conducted for 10 metabolites with and without clinical indicators to further assess the prognostic value of metabolites. Finally, three metabolites remained independent prognostic factors of overall survival. The correlations between the three metabolites and survival were similar in the combination model with or without clinical factors (**Figure 5A**). As we observed, a high level of MG(20:4)isomer (HR = 1.62) and low levels of 9,12-octadecadienoic acid (HR = 0.67) and L-isoleucine (HR = 0.56) correlated with poor overall survival in the single biomarker model.

We then calculated the risk scores for all patients using coefficient values of the three metabolites in the single biomarker model. Each patient was stratified into low- and high-risk groups by median of risk scores. As expected, patients in the high-risk group had worse survival than in the low-risk group as shown in **Figure 5B**. Furthermore, there was a trend that more deaths, a higher level of MG(20:4)isomer, and lower levels of 9,12-octadecadienoic acid and L-isoleucine were in the high-risk group compared with the low-risk group (**Figure 5C**).

### Retinol Metabolism and Linoleic Acid Metabolism as the Most Perturbed Metabolic Pathways

**Figure 6:**
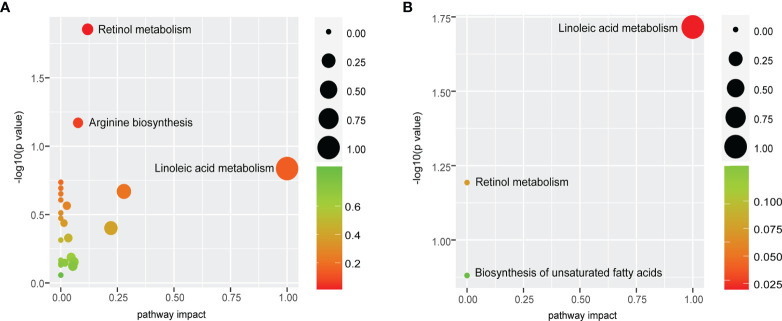
Retinol metabolism and linoleic acid metabolism are common features of ESCC patients. **(A)** Pathway analysis of commonly altered 105 metabolites from patients versus controls revealed that retinol metabolism was the most perturbed pathway. **(B)** Pathway analysis of 18 biomarkers displayed that the most significant pathway was linoleic acid metabolism. Pathway analysis module of MetaboAnalyst 5.0 was used to perform the pathway enrichment analysis (*y*-axis, enrichment *p*-values) and the pathway topology analysis (*x*-axis, pathway impact values indicating centrality and enrichment of a pathway). Circle color represents enrichment significance with darker color indicating a higher level of significance, and circle size correlates with pathway impact value of the pathway.

Differential metabolites were mapped to KEGG pathways using pathway analysis module of MetaboAnalyst 5.0. Pathway analysis of commonly altered 105 metabolites from patients versus controls revealed 23 tumor-related metabolic pathways including metabolism of cofactors and vitamins, amino acid metabolism, nucleotide metabolism, lipid metabolism, and carbohydrate metabolism. Among them, retinol metabolism was the most perturbed pathway (*p* < 0.05, **Figure 6A**) with upregulation of all-trans-13,14-dihydroretinol and downregulation of 11-cis-retinol and 4-hydroxyretinoic acid in the ESCC groups. Further pathway analysis of 18 biomarkers for early detection and prognosis revealed that linoleic acid metabolism was the most significant pathway (*p* < 0.05, **Figure 6B**) mapped by 9,12-octadecadienoic acid.

### Correlation Analysis of Serum and Tissue Metabolites

**Figure 7:**
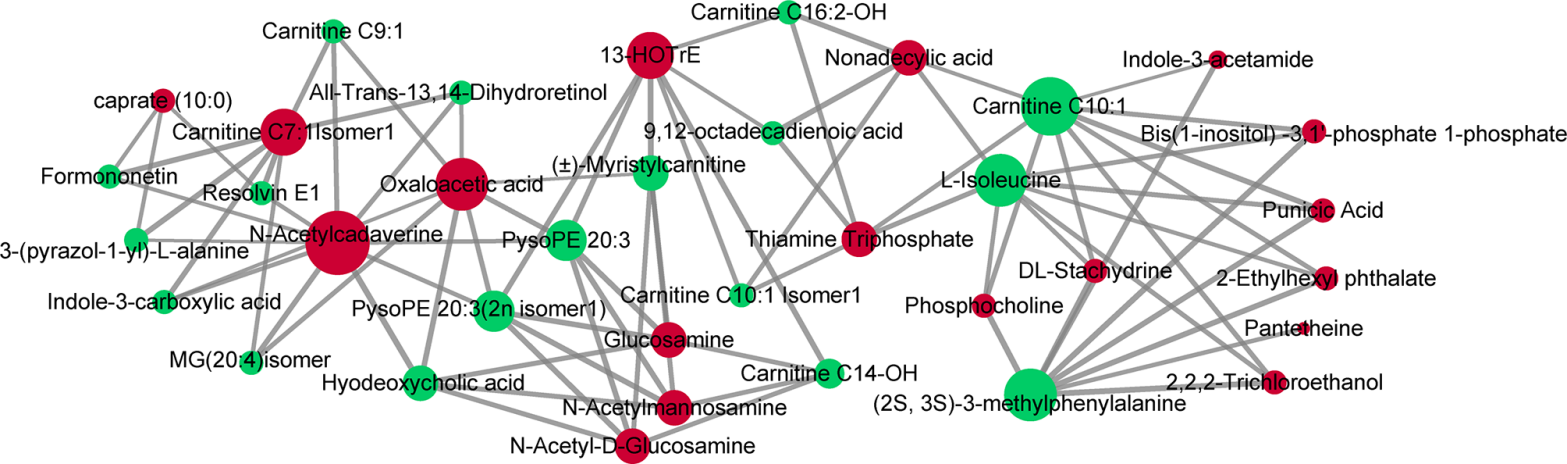
Correlations of serum and tissue metabolites. Pearson’s correlation between 18 serum biomarkers and 18 tissue differential metabolites. The nodes represent metabolites (serum metabolites are green and tissue metabolites are red). The line between two nodes indicates correlation scores (only correlation scores of >0.6 are kept) and thicker lines indicate stronger correlations.

Compared with adjacent normal tissues, 18 differential metabolites were identified (*p* < 0.05 and VIP > 1.5) in cancer tissues, consisting of 16 upregulated metabolites [glucosamine, punicic acid, 13-HOTrE, DL-stachydrine, *N*-acetylmannosamine, *N*-acetyl-D-glucosamine, 2-ethylhexyl phthalate, caprate (10:0), thiamine triphosphate, pantetheine, bis(1-inositol)-3,1′-phosphate 1-phosphate, *N*-acetylcadaverine, phosphocholine, 2,2,2-trichloroethanol, nonadecylic acid, and carnitine C7:1 isomer1] and 2 downregulated metabolites (indole-3-acetamide and oxaloacetic acid). We next performed Pearson’s correlation analysis to investigate the association between the 18 serum biomarkers and the tissue differential metabolites (**Figure 7**). This analysis showed strong positive correlations between the two groups of metabolites (*p* < 0.05 and *r* > 0.99). Specifically, PysoPE 20:3 and PysoPE 20:3(2n isomer 1) (glycerophospholipids) were positively related to *N*-acetylmannosamine and *N*-acetyl-D-glucosamine (sugar and its derivatives). (2S,3S)-3-Methylphenylalanine (amino acids) was positively associated with DL-stachydrine (organic acid and its derivatives), phosphocholine (nucleotide and its metabolites), bis(1-inositol)-3,1′-phosphate 1-phosphate (alcohols), and 2,2,2-trichloroethanol (alcohols). We also identified a positive correlation between (±)-myristylcarnitine (fatty acyls) and 13-HOTrE (oxidized lipids) and between hyodeoxycholic acid (bile acids) and *N*-acetylcadaverine (polyamines).

## Discussion

We conducted a multicenter, large-scale metabonomic study of esophageal cancer through UPLC-MS/MS. Our analysis revealed that patients with esophageal cancer exhibited distinctive metabolic characteristics compared with healthy controls by verification in different cohorts. Such metabolic alterations were likely attributed to dysregulation of multiple metabolic pathways. Significantly, we identified robust serum biomarkers for early detection and prognosis of esophageal cancer, offering an opportunity for the targeted screening of high-risk groups and individualized management.

We identified 105 serum metabolites as reliable metabolic profiles for patients with ESCC in the discovery and two validation cohorts, mainly relating to changes of amino acids and fatty acyls (acylcarnitines and glycerophospholipids). First, almost all amino acid-related metabolites were reduced, including glycine, serine, leucine, isoleucine, phenylalanine, tryptophan, glutamic acid, aspartic acid, alanine, arginine, histidine, and cysteine, except methionine metabolites. Similar findings have been observed in previous studies on both ESCC and EAC (esophageal adenocarcinoma) (15, 16). Amino acids are the most frequently reported altered metabolites in cancer (17), which relate to increased oxidative metabolism, gluconeogenesis, and energy production in cancer patients (18). Specifically, methionine cycle flux specifically influences the epigenetic state of cancer cells and drives tumor initiation (19), and the cross-talk between glucose and methionine regulates life span (20). Serine metabolism supports the methionine cycle and DNA/RNA methylation through *de-novo* ATP synthesis in cancer cells (21). Serine/glycine biosynthesis affects cellular antioxidative capacity, thus supporting tumor homeostasis (22). Altered branched-chain amino acid (BCAAs: valine, leucine, and isoleucine) metabolism has been implicated in cancer progression and the key proteins in the BCAA metabolic pathway serve as possible prognostic and diagnostic biomarkers in human cancers (23). Aromatic amino acids tyrosine, phenylalanine, and tryptophan represent potential biomarkers and relate to gastroesophageal cancer (24). Tryptophan metabolism through the kynurenine pathway (KP) is involved in the regulation of immunity, neuronal function, and intestinal homeostasis (25). Our observations that disorders of amino acid metabolism were common alterations in ESCC have important implications for further investigation into the relationship between metabolic alterations and carcinogenesis. Another major class of disordered metabolites was acylcarnitines in our study: that medium- to long-chain acylcarnitines (octanoylcarnitine, nonanoylcarnitine, decanoylcarnitine, undecanoylcarnitine, dodecylcarnitine, tetradecanoylcarnitine, hexadecadienoylcarnitine, stearidonyl carnitine) in ESCC patients were significantly decreased compared with controls. A previous study by Xu et al. also reported the downregulation of acylcarnitines (octanoylcarnitine, nonanoylcarnitine, decanoylcarnitine, and undecanoylcarnitine) in ESCC patients (26). Given that these acylcarnitines as the main substrates of mitochondrial lipid oxidation regulate energy balance through promoting ketogenesis and reducing protein consumption (27), the low levels can reflect alterations of the tricarboxylic acid cycle (TCA cycle) activity and β‐oxidation in patients with ESCC in the present study. We also observed alterations in the two groups of glycerophospholipids metabolites, LPE and LPC. Previous data demonstrated that significant alterations of LPE and LPC were in the serum of patients with esophageal squamous cell carcinoma, pancreatic ductal adenocarcinoma, liver cancer, and ovarian cancer (13, 28–31). These findings substantiated the diagnostic value of LPE and LPC. Of interest, benzene derivatives (xylene, ethylbenzene, and o-xylene) were elevated relatively in the serum of patients with ESCC in our study. Benzene overexposure strongly elevates the incidence of cancer and risks of mortality, through increasing oxidative damage and cytogenetic changes (32, 33). While understanding of the carcinogenicity of benzene derivatives in ESCC is limited, this finding resulted likely from the interaction between environmental and genetic factors.

One of our overarching goals was to identify metabolite-based biomarkers for early detection. Here, we trained diagnostic models using logistic regression after LASSO and random forests. A set of 15 metabolites was screened as novel diagnostic markers, including all-trans-13,14-dihydroretinol, (±)-myristylcarnitine, (2S,3S)-3-methylphenylalanine, 3-(pyrazol-1-yl)-L-alanine, carnitine C10:1, carnitine C10:1 isomer1, carnitine C14-OH, carnitine C16:2-OH, carnitine C9:1, formononetin, hyodeoxycholic acid, indole-3-carboxylic acid, PysoPE 20:3, PysoPE 20:3(2n isomer1), and resolvin E1. Importantly, our diagnostic biomarker panel performed excellently in the training and validation groups (test set, the entire discovery group, two independent validation cohorts) with accuracies more than 90%, which would be a very meaningful subject of our further study. Intriguingly, almost all of these have been previously associated with cancers. For instance, increased levels of all-trans-13,14-dihydroretinol, metabolites of vitamin A (all-trans-retinol) produced by retinol saturase (RetSat) (34, 35), result in an accelerated apoptosis induction through reduction of all-trans-retinoic acid (atRA) (36). Acylcarnitine, generated by mitochondrial metabolism of amino acids and fatty acids, has been implicated in mitochondria-mediated inflammation and cellular stress promotion (37). Medium-chain acylcarnitines (C6–C12) are positively associated with the risk of prostate cancer progression, while long-chain acylcarnitines (C14–C18) are inversely associated with advanced stages (38, 39). Past research found that octanoylcarnitine and decanoylcarnitine were closely correlated with the treatment effect of ESCC (26). (2S,3S)-3-Methylphenylalanine can prevent mitochondrial damage and reduce apoptosis of cells (40). The level of 3-(pyrazol-1-yl)-L-alanine is closely related to gastric cancers (41). Indole-3-carboxylic acid, a microbial tryptophan metabolite, can enhance tumor malignancy and suppress antitumor immunity by activating aryl hydrocarbon receptor (AHR) (42, 43). Formononetin (FMNT), a isoflavonoid, possesses anti-inflammatory, antioxidant, and antitumoral properties (44–46), and supplementation of isoflavonoids can reduce the incidence and mortality of cancers (47, 48). Hyodeoxycholic acid (HDCA) can suppress intestinal epithelial cell proliferation through the FXR–PI3K/AKT pathway (49). The reduced levels of lysophosphatidylethanolamine (LPEs), key components of cellular membranes, can explain the rapid cellular proliferation of malignancies (50). Resolvin E1 inhibits oxidative stress, autophagy, and apoptosis by targeting Akt/mTOR signals (51), suppresses tumor growth, and enhances cancer therapy (52). Furthermore, we detected higher levels of four downregulated metabolites [hyodeoxycholic acid, (2S,3S)-3-methylphenylalanine, carnitine C9:1, and indole-3-carboxylic acid] in I–IIA stage (early cancer) than in IIB–IV stage (advanced cancer), which could serve as predictive biomarkers of early cancer, in which decreasing levels were associated with increased tumor burden. In summary, this metabolite-based diagnostic panel would be effective tools for early screening and diagnosing ESCC patients from high-risk populations in China.

Here, potential prognostic predictors were explored by Kaplan–Meier log-rank tests and univariate and multivariate Cox regression analyses. Our findings indicated that MG(20:4)isomer, 9,12-octadecadienoic acid, and L-isoleucine remained prognostic biomarkers of overall survival for ESCC with similar results regardless of whether prognostic clinical factors were incorporated or not. It suggested that this model based on three indicators, which did not involve the clinicopathologic information of patients, could be easily used in clinical practice. MG(20:4)isomer was upregulated and correlated with poor prognosis in ESCC patients, while 9,12-octadecadienoic acid and L-isoleucine did the opposite. When patients were stratified into low- and high-risk groups based on this model, patients in the high-risk group tended to have lower rates of survival and more deaths. Similarly, previous studies have also reported associations of three metabolites with different malignancies. MG(20:4)isomer, namely, eicosanoic acid monoglyceride, an arachidonic acid derivative and canonical endocannabinoid, is an isomer of 2-arachidonoylglycerol (2-AG) and 1-arachidonoylglycerol (1-AG). Canonical endocannabinoids have anti-inflammatory and anticancer properties by activating cannabinoid receptors CB1 and CB2. One study reported that both 2-AG and the activity of 2-AG decomposing enzymes [catabolic enzyme monoacylglycerol lipase (MAGL)] were elevated in lung squamous cell carcinoma tissue compared with normal adjacent lung tissue (53). Another study suggested that treatment with mixed CB1/CB2 agonist WIN-55,212–2 resulted in inhibition of skin tumor growth (54). A study showed that 9,12-octadecadienoic acid, belonging to linoleic acid metabolism, was significantly increased in preoperative lung cancer patients compared with healthy volunteers and postoperative lung cancer patients (55). Significant alterations of linoleic acid metabolism have been observed in many other cancer types associated with inflammatory-mediated damage, immune response, and cell proliferation (colorectal cancer, bladder cancer, and renal cell carcinoma) (56, 57). Linoleic acid has also been reported as one of the biomarkers for the diagnosis (12, 13, 58) and therapeutic efficacy (59) of patients with ESCC. Interestingly, the model constructed by linoleic acid and other 11 differentiating metabolites showed good predictive values for distinguishing EAC, high-risk (BE and HGD), and control groups (16). L-Isoleucine affects cancer cell state as well as systemic metabolism in individuals with malignancy (60). The deficiency of L-isoleucine is one metabolic characteristic for patients with gastric cancer after chemotherapy, and the correction of this metabolic deficiency improves the life quality of patients (61). Together, our findings have potential important implications for therapeutic decision-making and risk stratification for the management of patients with ESCC.

The pathway analysis of 105 metabolites from patients versus controls also confirmed that dysregulation of amino acid metabolism, lipid metabolism, vitamin metabolism, nucleotide metabolism, and carbohydrate metabolism was related to ESCC. We identified retinol metabolism as the most perturbed pathway with elevation of all-trans-13,14-dihydroretinol and reduction of 11-cis-retinol and 4-hydroxyretinoic acid in cancer. Under normal circumstances, atRA is the most biologically active retinol metabolite binding to retinoic acid receptor α (RARα) playing important roles in cell differentiation, proliferation, and apoptosis (62). Normal RetSat catalyzes all-trans-retinol to atRA, otherwise to all-trans-13,14-dihydroretinol. Deficiency of atRA has proven to contribute to colon carcinogenesis, while returning to normal level reduces the risk of cancer (36). In addition, atRA not only inhibits angiogenesis and metastasis of ESCC though angiopoietin receptor Tie2 (63) but also induces apoptosis of metaplastic Barrett’s cells *via* p38 and caspase pathways (64). In the research of Barrett’s esophagus organotypic model, atRA alters the squamous cytokeratin profile of EPC2 toward a more columnar expression pattern (65). This suggests that RetSat in retinol metabolism pathway is emerging as a promising therapeutic target for ESCC, and atRA has a potential role in therapy and chemoprevention of patients with ESCC and Barrett’s esophagus. Importantly, linoleic acid metabolism was also identified as the most significant pathway in the pathway analysis of 18 biomarkers, which validated a similar observation from previous smaller studies of patients with ESCC (12, 13, 58) and EAC (16). Linoleic acid metabolism is mediated by cytochrome P450 (CYP1A2, CYP2C, CYP2J, CYP2E1, and CYP3A4) to proinflammatory and proangiogenic oxylipins resulting in tumor growth or metastasis (66). Previous studies suggested that 12,13-epoxyoctadecenoic acid (EpOME) as a metabolite of linoleic acid produced by CYP monooxygenase increased cytokine production and JNK phosphorylation *in vitro* and exacerbated AOM/DSS-induced colon tumorigenesis *in vivo*, which revealed CYP2C enzymes being a novel therapeutic target for patients with colon cancer (67). Together with a previous work, it is therefore speculated that cytochrome P450 will be a novel therapeutic target for ESCC and deserves further investigation.

Correlation analysis of serum and tissue metabolites in different samples from the same patient demonstrated that these molecules mainly belonged to amino acid and lipid metabolism. Although differences were observed, there were significant positive correlations between serum biomarkers and tissue differential metabolites. The decreases of PysoPE 20:3 and PysoPE 20:3(2n isomer 1) in serum were mainly due to the increasing consumption of LPEs for constituting cell membranes, which were consistent with the increases of *N*-acetyl-D-glucosamine and *N*-acetyl-D-glucosamine in tissue caused by high activity of nucleotide synthesis and cellular proliferation. (2S,3S)-3-Methylphenylalanine reacts with 2-oxoglutarate into L-glutamate and (3S)-2-oxo-3-phenylbutanoate by 2-oxoglutarate aminotransferase in the process of glutamate providing 2-oxoglutarate for TCA cycle (68). For its correlated tissue metabolites, for instance, DL-stachydrine, as a derivative of proline, can not only be degraded to but also synthesized from glutamate (69, 70). Increased transport of choline into cancer cells results in a high level of phosphocholine in tissues (a substance converted from choline *via* phosphorylation by choline kinase) thereby promoting cell growth and proliferation (71). Bis(1-inositol)-3,1′-phosphate 1-phosphate and CMP convert into CDP-1L-myo-inositol and inositol 3-phosphate in inositol phosphate metabolism providing second messengers in cellular signal transduction (72). Elevation of 2,2,2-trichloroethanol in tissue may be related to hyperactive metabolism of xenobiotics by cytochrome P450. Both the high level of 13-HOTrE in tissue and the low level of (±)-myristylcarnitine in serum were the result of dysregulation of lipid metabolism in cancer. In conclusion, although serum and tissue metabolites were quite different, they maintained strong metabolite correlations from tumor-derived metabolic disorders. It further suggested that these serum metabolites could serve as non-invasive biomarkers for patients.

The representativeness of the study population was ensured by multicenter, large-scale data for ESCC patients and normal controls. Detailed clinicopathological information and long-term follow-up minimized the influence of confounding factors on screening biomarkers. Validation in two independent cohorts could support further extension and application of these diagnostic biomarkers. Different combinations in multivariate Cox regression analyses confirmed the reliability and clinical utility of prognostic biomarkers. Nevertheless, some limitations should also be acknowledged. Targeted metabolomics analysis is necessary to further verify these serum metabolite biomarkers, and prospective larger cohorts are needed to validate prognostic biomarkers given the retrospective design and non-uniform follow-up.

Altogether, we revealed serum metabolic profiles for patients with ESCC using UPLC-MS/MS-based metabolomics technology. Novel serum metabolic diagnostic biomarkers could effectively distinguish esophageal cancer patients from healthy controls, offering an opportunity for the early detection and diagnosis of esophageal cancer patients in asymptomatic population. Moreover, prognostic biomarkers would provide a new direction for the risk-stratified management and individualized therapeutic decision-making for both patients and doctors. Significant metabolic pathways provide mechanistic insight into future targeted therapies.

## Data Availability Statement

The raw data supporting the conclusions of this article will be made available by the authors, without undue reservation.

## Author Contributions

LDW conceived and designed this study and obtained financial support. Subject recruitment and biological material collection in Henan Province were supervised by LDW and carried out by PPW, XS, XNH, RHX, RW, ZMF, MMY, KZ, LLL, LYL, YC, JJJ, and YZY. The following authors from the various collaborating groups undertook the collection of samples and data in their respective regions: SGG in the First Affiliated Hospital of Henan University of Science and Technology, FYZ in Anyang Tumor Hospital, JLR in the Second Affiliated Hospital of Zhengzhou University, XML in Hebei Provincial Cixian People’s Hospital, and XZW in Linzhou People’s Hospital. PPW, MXW, and LDW participated in the study design, discussion of results, and manuscript preparation. PPW, XS, XKZ, MXW, JFH, KZ, YJC, and JL performed data curation, statistical and bioinformatic analyses, and original draft preparation. All authors contributed to the article and approved the submitted version.

## Funding

The study was supported by grants from the 2016 doctoral program of the First Affiliated Hospital of Zhengzhou University (2016-BSTDJJ-03) and the National Natural Science Foundation of China (U1804262) to LDW.

## Conflict of Interest

The authors declare that the research was conducted in the absence of any commercial or financial relationships that could be construed as a potential conflict of interest.

## Publisher’s Note

All claims expressed in this article are solely those of the authors and do not necessarily represent those of their affiliated organizations, or those of the publisher, the editors and the reviewers. Any product that may be evaluated in this article, or claim that may be made by its manufacturer, is not guaranteed or endorsed by the publisher.
