# Supplementary material for: Serum Metabolomic Profiling Reveals Biomarkers for Early Detection and Prognosis of Esophageal Squamous Cell Carcinoma
Source: Front Oncol. 2022 Jan 28;12:790933. doi: 10.3389/fonc.2022.790933 (PMC8832491; doi:10.3389/fonc.2022.790933)
Supplement: Supplementary file 1 [file DataSheet_1.docx]

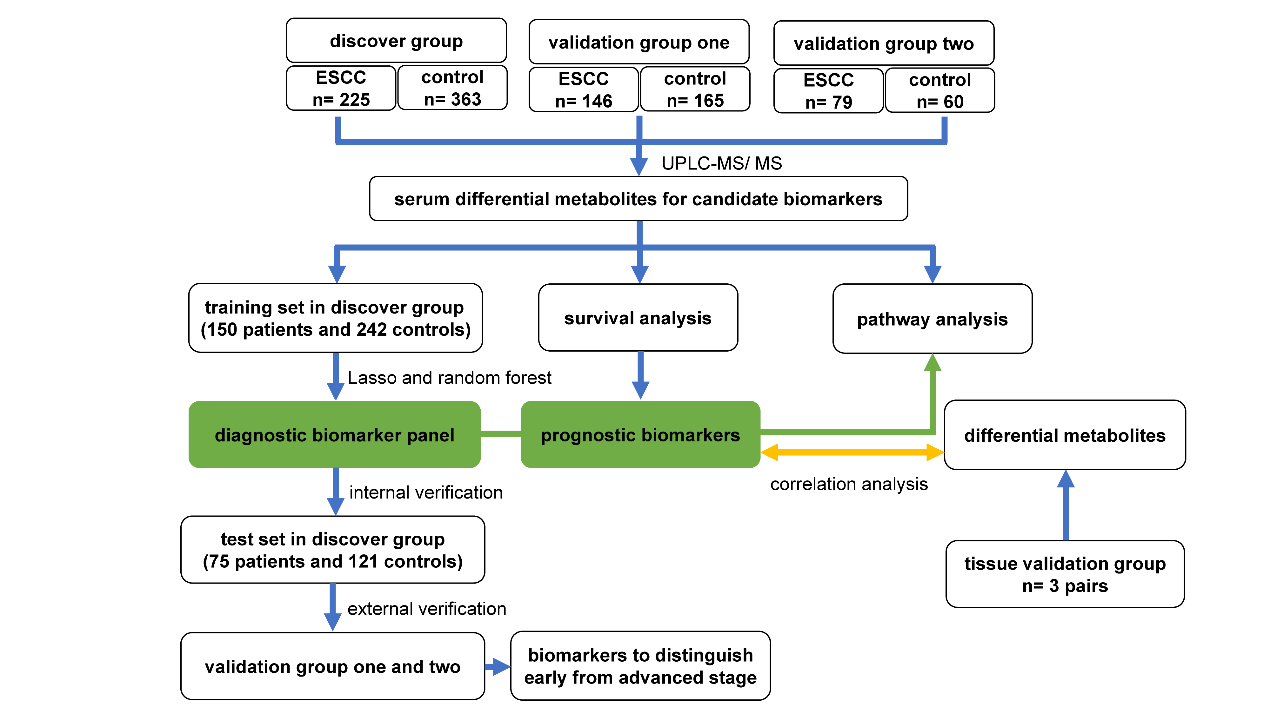


**Figure S1** Flow chart of study design.


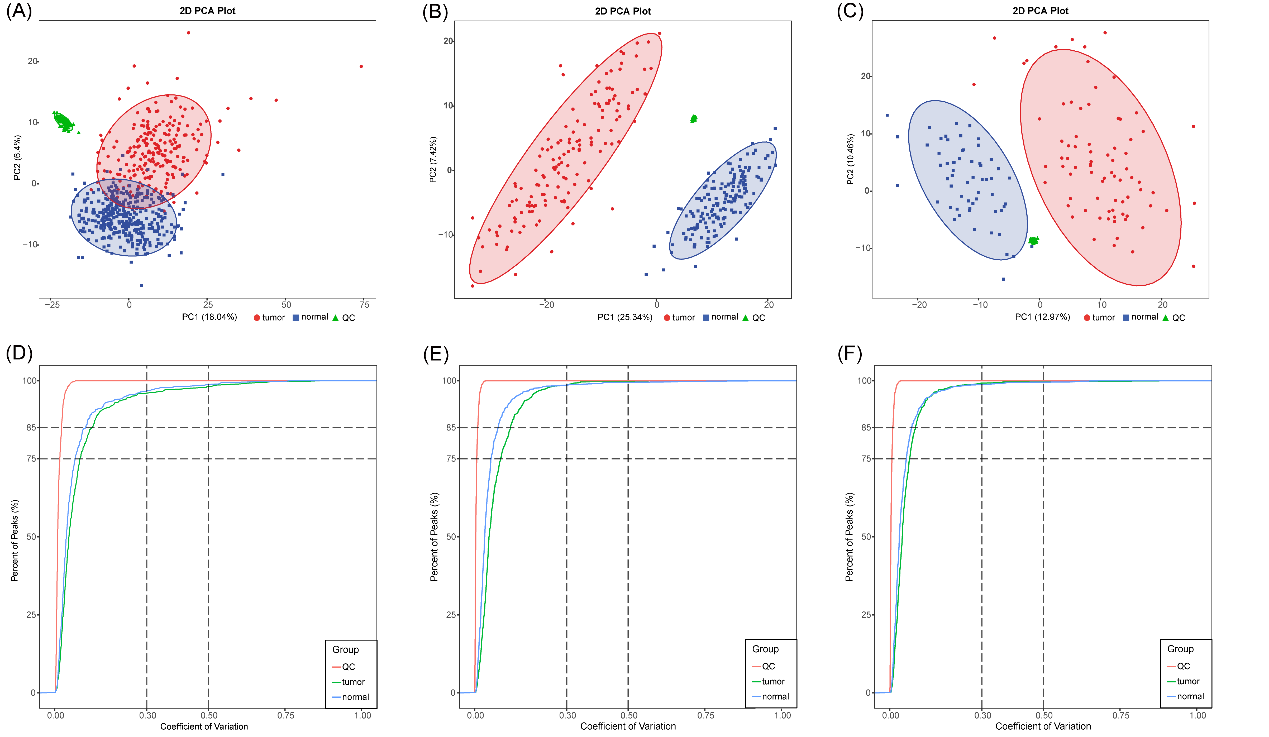


**Figure S2** Quality control of metabolomic data in three groups. (A-C) Principal component analysis (PCA) of metabolomic data including QC samples (mix) in discovery group (A), validation group one (B) and two (C): ESCC (red circles), normal (blue square) and QC (green triangles). (D-F) Coefficient of variation (CV) values are calculated by of metabolomic data including QC, tumor and normal samples in discovery group (D), validation group one (E) and two (F): ESCC (green), normal (blue) and QC (red). x axis: coefficient of variation, y axis: peak percentage of coefficient of variation.
